# HOXB13 promotes androgen independent growth of LNCaP prostate cancer cells by the activation of E2F signaling

**DOI:** 10.1186/1476-4598-9-124

**Published:** 2010-05-27

**Authors:** Young-Rang Kim, Kyung-Jin Oh, Ra-Young Park, Nguyen Thi Xuan, Taek-Won Kang, Dong-Deuk Kwon, Chan Choi, Min Soo Kim, Kwang Il Nam, Kyu Youn Ahn, Chaeyong Jung

**Affiliations:** 1 Department of Anatomy, Chonnam National University Medical School, Gwangju, Korea; 2 Department of Urology, Chonnam National University Medical School, Gwangju, Korea; 3 Department of Pathology, Chonnam National University Medical School, Gwangju, Korea; 4 Department of Statistics, Chonnam National University, Gwangju, Korea; 5 Research Institute of Medical Sciences, Chonnam National University, Gwangju, Korea

## Abstract

**Background:**

Androgen signaling plays a critical role in the development of prostate cancer and its progression. However, androgen-independent prostate cancer cells emerge after hormone ablation therapy, resulting in significant clinical problems. We have previously demonstrated that the HOXB13 homeodomain protein functions as a prostate cancer cell growth suppressor by inhibiting androgen-mediated signals. However, the role of the HOXB13 in androgen-independent growth of prostate cancer cells remains unexplained.

**Results:**

In this report, we first demonstrated that HOXB13 was highly overexpressed in hormone-refractory tumors compared to tumors without prostate-specific antigen after initial treatment. Functionally, in an androgen-free environment minimal induction of HOXB13 in LNCaP prostate cancer cells, to the level of the normal prostate, markedly promoted cell proliferation while suppression inhibited cell proliferation. The HOXB13-mediated cell growth promotion in the absence of androgen, appears to be mainly accomplished through the activation of RB-E2F signaling by inhibiting the expression of the p21^waf ^tumor suppressor. Indeed, forced expression of HOXB13 dramatically decreased expression of p21^waf^; this inhibition largely affected HOXB13-mediated promotion of E2F signaling.

**Conclusions:**

Taken together, the results of this study demonstrated the presence of a novel pathway that helps understand androgen-independent survival of prostate cancer cells. These findings suggest that upregulation of HOXB13 is associated with an additive growth advantage of prostate cancer cells in the absence of or low androgen concentrations, by the regulation of p21-mediated E2F signaling.

## Background

*Hox *homeobox genes contain highly homologous homeodomains and are considered transcription factors that regulate axial regional specification during embryonic development. Due to temporal and spatial colineality, *Hox *genes are expressed in a tissue-specific and frequently stage-related fashion. The Hox-13 paralog is especially important to the development of accessory sexual organs, including the prostate gland [1-3]. *Hoxb13 *has been shown to have limited expression at the caudal extent of the spinal cord, tail bud and urogenital sinus in an androgen-independent manner [4,5]. The expression of HOXB13 is restricted but abundant in the adult human prostate, representing almost one out of 2,000 transcripts [6]. However, the role of HOXB13 during the adult stage is mostly unknown. Mice homozygous for Hoxb13 loss-of-function mutations have shown overgrowth in all major structures derived from the tail bud [7] and malformation of the ducts of the ventral prostate, including complete loss of secretory proteins [8]. Hoxb13 mutant mice do not develop a prominent phenotypical change but do have a swollen prostate in old mice, probably due to the functional redundancy of other Hox-13 molecules.

Prostate cancer (PCa) is the most commonly diagnosed cancer in North American men; a significant number of patients manifest androgen resistant PCa, leading to a fatal disease. Androgen receptor (AR) activity is essential to the growth and progression of PCa at all phases of the disease [9,10]. Therefore, androgen ablation therapy is the frontline treatment for metastatic PCa; however, the clinical response is transient, resulting in the recurrence of tumors. These androgen-independent (AI) tumors paradoxically depend on the AR [11,12], suggesting that AR-mediated signaling is required for the growth of cancer cells, even when absent or present only in low concentrations. However, the regulation of AR target genes under androgen-deprived conditions is not fully understood. There is evidence to support a link between the role of HOXB13 and the malignant progression of PCa.

The highly prostate-specific HOXB13 is involved in the formation and maintenance of this hormone-dependent organ [5,6,13,14]. In our previous studies, HOXB13 expression was correlated with the AR in both cultured PCa cells and PCa xenograft models. All PCa cell lines retaining the AR expressed moderate to high levels of HOXB13 whereas AR-negative cells had low to undetectable amounts of HOXB13. However, the expression of HOXB13 and AR was not mutually regulated [5,15]. There is also close functional and mechanistic communications between HOXB13 and AR signaling. HOXB13 suppresses androgen-stimulated AR activity by physical interaction with the AR and subsequently inhibits the growth-regulating function of AR [15]. Due to the multifocal nature of PCa, however, the pattern of expression of HOXB13, during prostate tumorigenesis, has not been well defined [15,16]. These results suggest that the balance between the HOXB13 and the AR (and/or hormone response) may be important in the development and progression of PCa cells. In this study, we investigated the pattern of expression of HOXB13 in androgen-dependent (AD) and AI tumors and the functional and mechanistic role of HOXB13 in hormone-refractory PCa cells.

## Results

### Anti-HOXB13 antibodies

**Figure 1:**
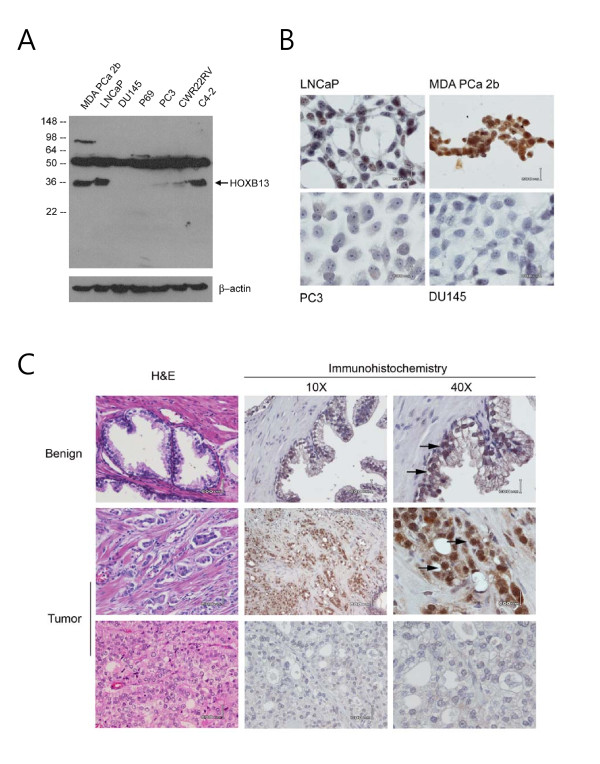
**Validation of HOXB13-specific antibodies in prostate cancers**: Expression of HOXB13 in various PCa cells by Western blot analysis (A) and immunocytochemistry (B). While LNCaP and MDA PCa 2b cells were HOXB13-retained cells, PC3 and DU145 cells were HOXB13-deficient cells. HOXB13 was immunostained in prostates with normal or tumor tissue with routine hematoxyline-eosin staining. Arrows indicate HOXB13 localized to the nucleus of luminal epithelial cells or cancer cells.

To study the tissue distribution and expression pattern of HOXB13 protein in PCas, HOXB13-specific antibodies are critical tool. To date, there are no anti-HOXB13 antibodies suitable for immunohistochemistry that can be purchased in the commercial market. Therefore, anti-HOXB13 antibodies were developed and their specificity to HOXB13 was tested by Western blot analysis using various PCa cell lines. As shown in Figure 1A, anti-HOXB13 antibodies had a band at 35kDa, which was highly expressed in the LNCaP, C42, and MDAPCa2b cells, with only minor to no expression in the PC3, DU145, P69, and CWR22RV cells. These results were consistent with the expression pattern of *HOXB13 *RNA as previously demonstrated [15]. These anti-HOXB13 antibodies did not react with recombinant homologous HOXA13 or HOXD13 in transfection experiments (data not shown). However, the antibodies nonspecifically reacted to protein around 50kDa. To confirm that the antibodies could be used to localize HOXB13 in tumor cells, immunocytochemistry was performed in both HOXB13 positive and negative cells (Figure 1B). HOXB13 was detected in the nuclear compartment of HOXB13-positive LNCaP and MDAPCa2b cells, but not in the HOXB13-negative PC3 and DU145 cells; these findings suggested that antibodies did not react to the native form of the 50 kDa protein observed in the Western blot. Next, to evaluate the tissue expression of HOXB13 in PCas, immunohistochemistry was performed on formalin-fixed and paraffin-embedded PCas (Figure 1C). Similar to the results of prior mouse studies [4,5], moderate expression of HOXB13 was observed in benign prostate luminal epithelium, which was confined to the nuclear compartment (arrows in upper row). In localized PCas, some ductal epithelial cells highly expressed HOXB13 (arrows in middle row) while some ducts did not (lower row), probably due to the multifocal and heterogeneous nature of PCa.

### HOXB13 was overexpressed in androgen-refractory prostate cancer

**Figure 2:**
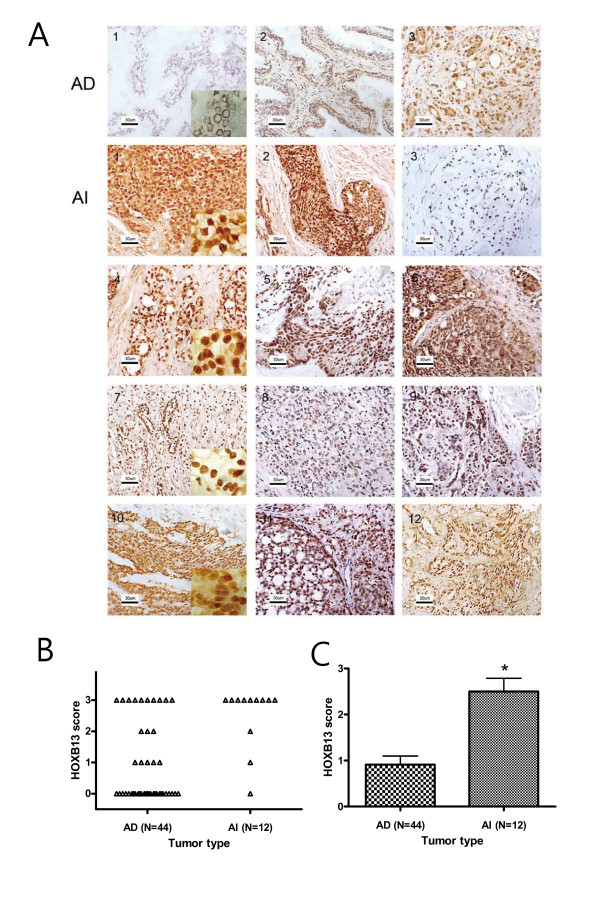
**Expression of HOXB13 in hormone-refractory prostate cancers**: *A*, HOXB13 was immunostained in both androgen-dependent (AD) and androgen-independent (AI) tumors. AI tumors were acquired through transurethral resection of the prostate. High-powered pictures are shown in inlets of some selected figures. *B*, Dot plot demonstration of HOXB13 scores from each of the AD and AI tumors. Expression of HOXB13 in each tumor was scored (0, absent; 1, weak; 2, intermediate; and 3, strong). C, scores of HOXB13 are shown in the bar graph. The *p *value was determined by Pearson's chi square statistics with a value of 11.2707. *. *p *= 0.0008.

The expression of HOXB13 was investigated in tumor specimens from failed androgen ablation therapy and compared to tumors without PSA recurrence. Fifty six clinical samples of hormone-dependent (n = 44) and hormone-refractory (n = 12) PCas were successfully stained. Among AD tumors, 31 (70%) showed negative or weak staining (score 0-1) and only 13 (30%) had moderate or strong staining (score 2-3) for HOXB13. In Figure 2A, AD1 represents no HOXB13 expression while AD2-3 represents moderate to high expression of HOXB13. Although there were some difficulties in the precise scoring of the HOXB13 expression due to the heterogeneous population of PCa, expression of HOXB13 in the AD tumors were predominantly low. Of note was that the AD tumors with high Gleason scores tended to have high expression of HOXB13 (data not shown). For the hormone-refractory tumors, however, 10 (83%) samples showed moderate to strong expression while only 2 (17%) were negative or weak. At least 50% of tumor cells overexpressed HOXB13 in the 10 samples. All 12 AI samples are shown in Figure 2A AI 1-12. Figure 2B shows the distribution of the HOXB13 expression scores in the androgen independent and androgen dependent tumors. Overall, hormone-refractory tumors showed significantly stronger expression of HOXB13 than androgen dependent tumors (Pearson's = 11.2707, *p *= 0.0008) (Figure 2C), suggesting that HOXB13 expression is somehow altered, or that high HOXB13 expressing cells are selected during androgen-independent progression of PCa.

### Alteration of HOXB13 expression in LNCaP prostate cancer cells

**Figure 3:**
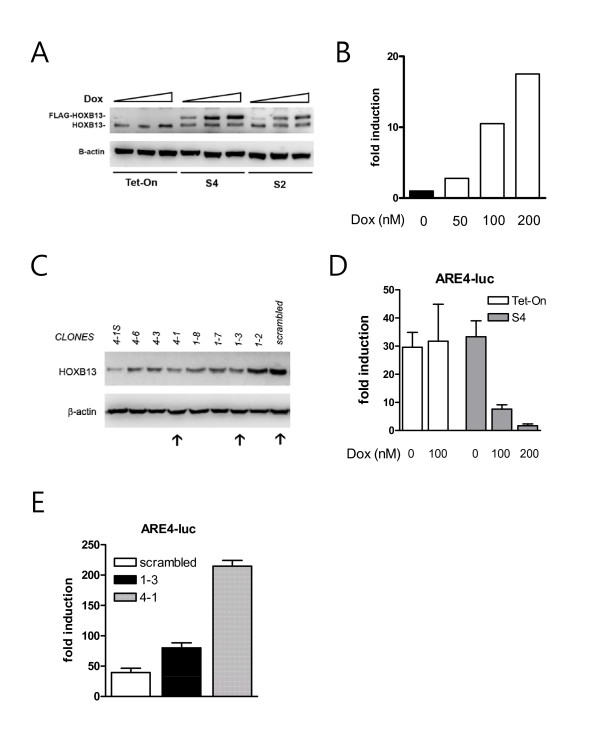
**Construction of HOXB13-manipulated LNCaP prostate cancer cells**: *A*, HOXB13 was induced by doxycycline in S2 and S4 cells compared to no expression in the Tet-on control cells. *B*, Recombinant HOXB13 in S4 the LNCaP cells was quantitated by densitometry. The endogenous HOXB13 level was arbitrarily set as one fold. *C*, Western blot analysis showed clones of HOXB13-suppressed LNCaP cells in addition to scrambled DNA-transfected LNCaP. *D-E*, By the reporter transcription assay, the effect on androgen-stimulated AR activity was tested in the Tet-on and S4 cells and HOXB13-suppressed clones. Cells were transiently transfected with 100 ng of pGL-ARE4-Luc and 2 ng of ranilla with or without 10 nM R1881. When needed, doxycycline was added with R1881 to induce HOXB13. Luciferase assays were performed 48 h post-transfection. Values indicate fold induction (RLU with androgen/RLU without androgen). Each *bar *represents the mean ± S.D.

In our previous studies [15,17], we found that constitutive expression of HOXB13 drove PCa cells to cell death. To avoid unwanted xenotoxic effects caused by HOXB13, we have developed HOXB13-inducible cell lines using the Tet-On system (BD Biosciences). After selected by antibiotics (600 μg/ml G418 and 1 μg/ml puromycin) and ring cloning, the two best LNCaP clones with doxycycline (Dox)-inducible HOXB13 were established. As shown in Figure 3A, HOXB13 was demonstrated by Western blot analysis in HOXB13-inducible LNCaP cells, named S2 and S4, in addition to Tet-on control cells. FLAG-tagged HOXB13 was gradually increased by Dox in a dose-responsive manner (50, 100, 200 nM) while the level of endogenous HOXB13 expression was not significantly altered by Dox. To maintain the overexpressed HOXB13 at physiological levels, the concentration of Dox required to induce recombinant HOXB13, to the level of normal prostate, was determined. LNCaP cells express only 10% of HOXB13 compared to the normal prostate [15]. Densitometric quantitation of FLAG-HOXB13 demonstrated that the FLAG-HOXB13 was induced ten-fold at concentrations of 100 nM Dox (Figure 3B). The Dox concentration was maintained at 100 nM for further analyses. Suppression of HOXB13 in the LNCaP cells was achieved using a retroviral vector system. As shown in Figure 3C, several stable transfectants were selected. These included 1-3 and 4-1 in addition to cells transfected with scrambled DNA. Clone 4-1S was not expandable. Next, to validate whether the established clones retained known biochemical properties, a reporter transcription assay was performed to determine the AR repressive function of HOXB13. HOXB13 was previously shown to strongly repress androgen-stimulated AR activity [15]. HOXB13-inducible cells (S4) under the influence of Dox showed dramatic inhibition of ARE4, a synthetic promoter containing four copies of androgen response elements, in a dose responsive manner (Figure 3D). S2 cells showed the same phenomenon (data not shown). Tet-on control cells did not alter the androgen-stimulated AR activity by the addition of Dox. At the same time HOXB13-suppressed cells showed dramatic promotion of androgen-stimulated AR activity (Figure 3E).

### HOXB13 promotes androgen-independent prostate cancer cell growth

**Figure 4:**
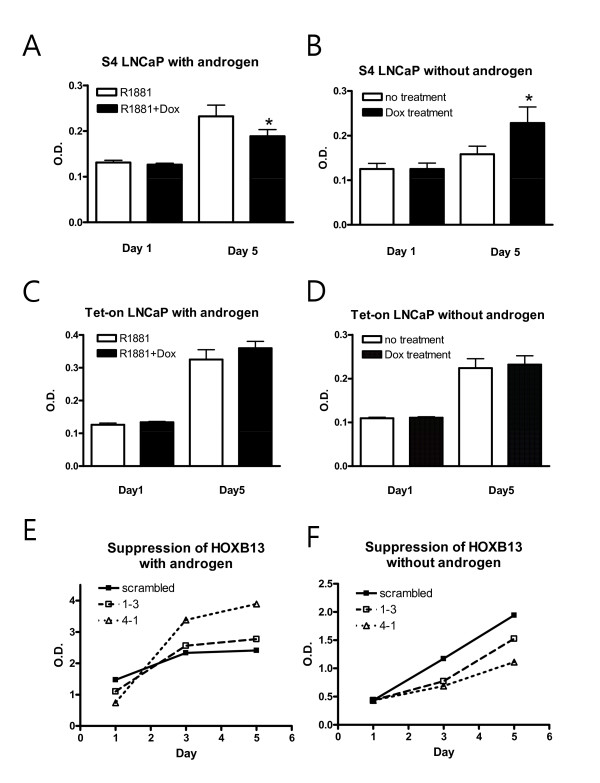
**Effect of HOXB13 on androgen-independent prostate cancer cell growth**: S4 and Tet-on cells were grown under androgen-rich conditions (A, C) or androgen-deprived conditions (B, D) for up to 6 days in 100 nM doxycycline. HOXB13-suppressed cells (1-3 and 4-1) with scrambled control cells were also grown under similar conditions (E, F). The cells were stained with MTT reagent and measured at an optical density at 570 nm. The * in the diagram, determined by a two-tailed Student's *t *test, corresponds to the difference between the two groups (*p *< 0.05). In E and F, repeated-measures ANOVA revealed very significant interaction effect between groups and days (p < 0.0001).

To monitor the *in vitro *cell growth of the HOXB13-manipulated cells, a MTT assay was performed. In the presence of the synthetic androgen (R1881) at a final concentration of 1nM, induction of HOXB13 by Dox drove the S4 cells to growth inhibition as previously observed (Figure 4A). On the other hand, in the absence of androgen, induction of HOXB13 conferred a positive growth signal on the S4 cells (Figure 4B). Addition of Dox did not affect cell growth of the Tet-on cells in both circumstances (Figure 4C-D). Growth assays in HOXB13-suppressed LNCaP cells were also studied; they showed that inhibition of HOXB13 expression promoted cell growth in the presence of androgen (Figure 4E) and inhibited cell growth in the absence of androgen (Figure 4F). In both circumstances, repeated-measures ANOVA revealed very significant interaction effect between groups and days (p < 0.0001). This phenomenon explains why HOXB13 is overexpressed in androgen-refractory tumors and also suggests that HOXB13 is involved in PCa cell proliferation in both the androgen dependent and independent environment.

### HOXB13 inhibits expression of p21^waf ^in the absence of androgen

**Figure 5:**
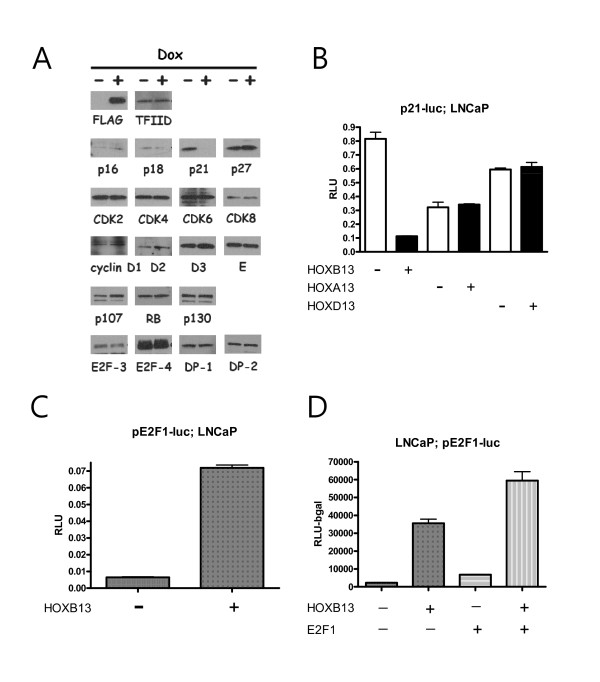
**Effect of HOXB13 on the p21-RB-E2F signaling pathway**: *A*, S4 cells were grown under hormone-deprived conditions for 3 days and induced for HOXB13 with 100nM doxycycline. Extracted lysates were evaluated for Western blot analysis. LNCaP cells were grown under hormone-deprived condition for 3 days. *B, *Cells were transfected with p21-luc and pFLAG-HOXB13, pCDNA-HOXA13, or pCMV-HOXD13. *C*, Cells were transfected with pE2F1-luc and pFLAG-HOXB13. *D*, Cells were transfected with pE2F1-luc, pFLAG-HOXB13, and pCDNA-E2F1. Corresponding control vectors were used to match each DNA. After 48 hours, the cells were evaluated by luciferase assays. Each *bar *represents the mean ± S.D.

Previously, we demonstrated that overexpression of HOXB13 caused G1 cell cycle arrest in AR-negative and HOXB13-deficient PC3 PCa cells [17]. HOXB13-induced cell cycle arrest appears to be due to the reduction of cyclin D1 and c-myc, which are targets of β-catenin/TCF signaling. However, HOXB13's effect on TCF target genes is minimal in AR-positive and HOXB13-retained CWR22RV PCa cells. In order to verify whether there are any altered proteins involved in the G1 cell cycle by HOXB13, HOXB13-inducible LNCaP cells (S4) were grown in charcoal dextran-treated (CDT)-FBS for 3 days in the absence of androgen. These cells were then treated with 100nM Dox for 48 hours and whole cell lysates were analyzed by Western blot. As shown in Figure 5A, HOXB13 dramatically inhibited expression of the p21^waf ^protein whereas HOXB13 did not affect expression of other cell cycle inhibitors, cyclins, and cdks. At the same time, HOXB13 did not alter the expression of downstream effectors of the p21^waf ^protein, including RB-related pocket proteins and E2F transcription factors. E2F1-2 is not shown in the figures due to the poor antibody reactivity. Next, the p21 promoter-derived luciferase assay was used to determine whether HOXB13 inhibits p21^waf ^at the transcriptional level. Figure 5B shows that HOXB13 decreased p21 promoter activity down to 15%, suggesting that HOXB13 inhibited transcription of p21^waf^. Repression of p21^waf ^was unique to HOXB13 since other HOX-13 paralogs, generously expressed in the prostate and possessing similar nucleotide sequences, did not suppress p21^waf ^transcription. To determine whether HOXB13-induced repression of p21^waf ^could activate the E2F1 response, the E2F1 promoter based luciferase assay was performed in the LNCaP cells. E2F1 is known to activate its own promoter by direct binding [18]. HOXB13 dramatically promoted the E2F1-mediated response (Figure 5C) and additional E2F1 showed synergistic effects (Figure 5D, two-way ANOVA: F = 12.44, p < 0.01). These results suggest that HOXB13 is involved in the activation of the oncogenic E2F signal by downregulation of p21^waf ^expression.

### Promotion of the E2F signal by HOXB13 was mainly due to p21^waf ^repression

**Figure 6:**
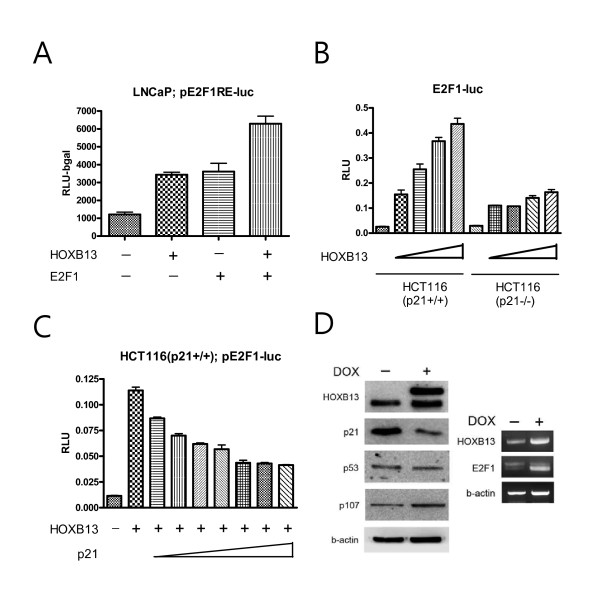
**Effect of HOXB13 on p21-dependent E2F1-mediated transactivation**: *A*, LNCaP cells were grown under hormone-deprived conditions for 3 days and transfected with pE2F1(3X)-luc, pFLAG-HOXB13, and pCDNA-E2F1. *B*, Both HCT116 (p21+/+) and HCT116 (p21-/-) cells were transfected with pE2F1-luc and an increased amount of pFLAG-HOXB13. *C*, HCT116 (p21+/+) cells were transfected with pE2F1-luc, pFLAG-HOXB13, and an increased amount of pFLAG-p21. Corresponding control vectors were used to match each DNA. After 48 hours, the cells were evaluated for luciferase assays. Each *bar *represents the mean ± S.D. *D*, S4 cells were treated with Dox to induce HOXB13 followed by Western blot and RT-PCR analyses.

Since HOXB13's effect on E2F did not necessarily come from the regulation of the expression of p21^waf^, we tested whether HOXB13 affects E2F response elements by way of p21^waf^. First, we tested whether HOXB13's effect on the E2F1 promoter was mediated through the E2F1 binding sites. By using three copies of simple E2F1 response elements, HOXB13 and E2F1also showed additive effects of the E2F1 response (Figure 6A), suggesting that E2F1 promotion by HOXB13 was mediated by the E2F1 response elements. Next, we studied HCT116 colon cancer cells with either wild type or deleted p21^waf^. Both cells were transfected with pE2F1-luc and HOXB13. While HCT116 (p21+/+) showed a dramatic increase in E2F activity, in a dose responsive manner (up to 14 fold increase), HCT116 (p21-/-) cells showed minimal promoting effects (up to 4 fold increase) (Figure 6B). In addition, increase of p21 gradually decreased the E2F activity stimulated by HOXB13 in a dose responsive manner in the HCT116 (p21+/+) cells (Figure 6C). At maximum expression of p21, however, the activity did not completely return to basal levels. These results suggest that there is a minor p21-independent activation of E2F1 by HOXB13. To verify the alteration of the E2F1 target genes by HOXB13, Western blot and RT-PCR analyses were performed (Figure 6D). HOXB13 up-regulated expression of the p107 pocket protein, which is a target of E2F-mediated transactivation [19]. Since E2F1 protein expression is known to be barely detectable in cells grown under hormone-deprived conditions [20], RT-PCR analysis showed that HOXB13 stimulated expression of E2F1. These results suggest that HOXB13's promoting effect on E2F signaling mainly comes from a p21-dependent pathway.

## Discussion

HOXB13 is a highly specific prostate homeobox protein and is involved in the formation of this hormone-dominant organ [5,7,8,15]. Functionally, Hoxb13 loss-of-function mice show swollen prostate glands [8]. Involvement of HOXB13 during carcinogenesis of human tissues has been reported by many groups [21-24]. Many reports have published findings supporting a link between and several solid tumors, including breast cancer, cervical cancer, ovarian cancer, and skin cancers [16,24-29]. In breast cancer, HOXB13 was found to be overexpressed in hormone-refractory tumors compared to hormone-responsive tumors, further suggesting that HOXB13 might be a useful prognostic marker for hormone-refractory breast cancers [26]. In addition, the forced expression of HOXB13 promoted the growth of MCF10A estrogen receptor (ER)-negative breast cells along with increased migration and invasion. In this report, we have demonstrated that HOXB13 was also overexpressed in hormone-refractory prostate tumors; its expression provided a positive growth signal in the absence of androgen. Our previous reports showed no expression of HOXB13 in only PC3 and DU145 cells [15]. While these cells are described as androgen-independent prostate cancer cells, they are generally considered as 'non-prostate-like cancer cells' mainly due the deficiency of androgen receptors (AR). In addition, there was positive correlation between HOXB13 and AR with consequent functional counteraction between these two proteins, suggesting that HOXB13 functions as a PCa cell growth suppressor in the presence of androgen [15]. HOXB13 antagonizes the function of the androgen-stimulated AR signal by disrupting coactivator formation. Although the prognostic value of the HOXB13, for the progression of PCa, requires further study, it is surprising that the function of HOXB13 is paradoxically opposite, depending on the status of androgen. In fact, tumor suppressor NKX3.1 homeodomain protein is overexpressed in metastatic prostate cancer [30]. In ovarian cancer, HOXB13 promotes cancer cell proliferation and progression [28]. Taken together, the balance between HOXB13 and the AR is important for the modulation of growth in PCa cells. In other words, HOXB13 expression must be deregulated for the survival of androgen-independent tumors while its expression is highly maintained in tumors with high androgen responsiveness.

Some HOX proteins are involved in the progression of PCa. Both HOXC6 and HOXC8 have been shown to be overexpressed in more advanced metastatic and recurrent PCa [31,32]. Despite the high expression of HOXB13 in the normal prostate, alteration of HOXB13 expression during the transformation process remains controversial. Some studies suggest overexpression of HOXB13 in prostate tumors [13,33], while others reported no such change [15,34]. Using total RNA we did not observe a differential expression of HOXB13 between the benign and malignant tumors; this was likely due to the multifocal nature of PCa [15]. In addition, immunohistochemical staining did not demonstrate alteration of HOXB13 during the tumorigenic process (unpublished data). Suppression or down-regulation of HOXB13 has been observed in other tumors, including colorectal cancer and skin tumors [22-24,35], although the level of HOXB13 protein was not detectable in normal skin. It is believed that the expression pattern and role of HOXB13 can be significantly different in breast cancers and PCa based on the following observations: 1) Breast tissues are HOXB13-negative as reported by many groups [6,14-16]. Accordingly, all cultured breast cancer cells, including several MCF7, MCF10a, T47D, and MDA 231, showed no expression or only minimally expressed HOXB13 [16,36]. 2) While HOXB13 and estrogen signals can be mutually regulated in breast cancer [21], there is no mutual regulation of HOXB13 and the androgen receptor in PCa [15]. 3) HOXB13 promotes growth of MCF10A cells, ER-negative normal breast cells [26] but HOXB13 inhibits AR-negative PC3 PCa cells by regulating β-catenin/TCF signals [17]. Therefore, the role of HOXB13 is variable depending on the cell type itself and the cell environment.

**Figure 7:**
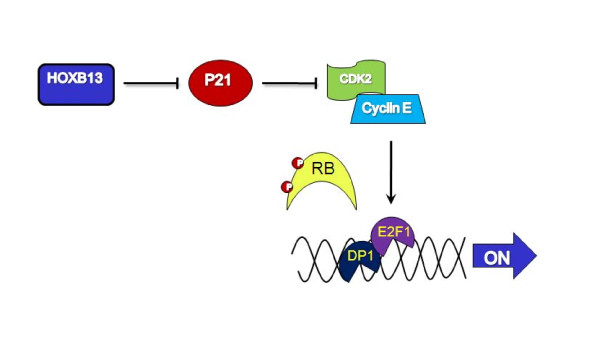
**Model of HOXB13-mediated regulation of E2F signaling**: Overexpression of HOXB13 in androgen-refractory prostate tumors inhibits the expression of the p21 ^waf ^tumor suppressor and subsequently activates cyclin dependent kinase 2 (CDK2) activities. Hyperphosphorylated RB releases E2F transcription factor, which drives the genes involved in cell proliferation, and results in increased cell survivability in the absence of hormone.

This study demonstrated that HOXB13 was overexpressed in androgen-refractory prostate tumors and was involved in providing a positive growth signal to PCa cells in the absence of androgen. In fact, Hoxb13 regulatory elements have been shown to successfully mediate transgene expression in castrated transgenic mice, demonstrating that HOXB13 expression is not affected by androgen or hormone blockage therapy [37]. Hormone-independent growth promotion by HOXB13 appears to be mainly due to the inhibition of tumor suppressor p21^waf ^expression and the consequent release of the E2F1 transcription factor from the RB family pocket proteins. More available E2F1 activates its target gene transcription, generally causing heperproliferation of cells [38,39]. The RB/E2F pathway is deregulated in many cancers [40,41] and overexpression of E2F1 also prevents androgen-withdrawal-mediated growth arrest and high levels of E2F1 expression represses neuroendocrine differentiation of androgen independent cells [20]. Taken together, a model of HOXB13's role in androgen-independent survival of PCa cells is shown in Figure 7. With low or no androgen influence, HOXB13 inhibits p21 expression to promote activity of CDK2. Consequent phosphorylation of the RB protein by CDK2 releases the E2F1 transcription factor to drive expression of its target gene, such as c-myc, p107, and E2Fs. These oncogenes provide positive growth signals to the PCa cells for survival in a harsh environment such as one without androgen hormones. The role of HOXB13 in androgen independent survival signaling pathways, such as interlukin-6 and protein kinase A-dependent CREB pathways, requires further study. In addition, the detailed mechanism of the action of HOXB13 on the p21 promoter requires further clarification to fully understand HOXB13's role in the p21-RB-E2F signaling pathway.

## Conclusions

Lack of information on androgen-independent progression of prostate cancer cells makes clinicians to difficult to properly treat patients with hormone-refractory cancers. This study demonstrated that HOXB13 homeodomain protein is overexpressed in hormone-refractory prostate cancer. Underlying mechanism on HOXB13-mediated growth stimulation seems to come from the specific inhibition of p21 followed by subsequent activation of RB/E2F1 signaling. This study presents a novel pathway to better understand androgen-independent survival of prostate cancer cells and suggests that upregulation of HOXB13 confers additive growth advantage to prostate cancer cells in no or low androgen status by alteration of p21-mediated E2F signal, a decisive growth regulator.

## Methods

### Patient cohort

Paraffin-embedded human primary PCas were obtained from patients after they provided informed consent to participate in this study at Chonnam National University Hospital between 1997 and 2005. Specimens from 56 patients were included; 12 had recurrent PCas with prior hormone adjuvant therapy [See Additional file 1; Table S1]. The treatment modalities were; orchiectomy, luteinizing hormone-releasing hormone analogue, estrogen, and combined androgen blockade. The median time from onset of the androgen ablation to androgen-independent progression was 15.6 months (range: 7-48). Due to the rarity of hormone-dependent tumors following hormone ablation therapy, the hormone-dependent tumors were acquired by radical prostatectomy while hormone-refractory tumors were obtained by transurethral resection of the prostate [See Additional file 2; Table S2]. Hormone-dependent tumors were defined as tumors with no recurrence of a PSA >0.4ng/ml after surgery. All cases had clinical follow-up for at least 3-years and up to 10 years. Benign prostate tissues obtained from radical prostatectomy procedures were used to determine the baseline expression of HOXB13.

### Antisera

Anti-HOXB13 antibodies were produced against a KLH fusion protein containing the C-terminal end of the human HOXB13 sequence (EKKVLAAKVKNSATP) in two New Zealand white rabbits as described by Komuves et al [24]. The synthetic peptides did not show any sequence homology to any known proteins searched for using BLAST. Antisera were produced under contractual agreement by Sigma Genosys (St. Louis, MO, USA). Antibodies were positively affinity purified by affinity chromatography against immobilized HOXB13 synthetic peptides.

### Immunostaining

For the immunocytochemistry, cells were plated onto 2-well chamber slides at 30% confluence. After overnight incubation, the cells were fixed with methanol/acetone solution for 15 minutes. Then, the cells were treated with 0.2% Triton X100 in PBS for 10 minutes. Anti-HOXB13 antibodies in 2% BSA/PBS were added to the slides for 1 hour. After washing, the slides were incubated with donkey anti-rabbit IgG (Fab fragment) conjugated to peroxidase (Jackson Laboratories). Colorimetric signals were detected using diaminobenzidine (DAB). Sections were counterstained with hematoxylin for microscopic evaluation. Immunohistochemistry was performed as described previously [24]. Tissues were deparaffinized followed by microwave antigen retrieval in citrate buffer. Endogenous peroxidase activity was destroyed by the treating tissue sections with 3% H_2_O_2_. After nonspecific reactivity was sequentially blocked by an Avidin-Biotin Blocking Reagent and 10% normal goat serum, the tissues were incubated with anti-HOXB13 antibodies and then with donkey anti-rabbit IgG (Fab fragment) conjugated to peroxidase (Jackson Laboratories). Colorimetric signals were detected using DAB. Sections were counterstained with hematoxylin for microscopic evaluation. Anti-androgen receptor antibodies (Upstate) were used to confirm tissue integrity. For the negative control slide, non-immune rabbit IgG was used. The immunostained slides were evaluated by two different investigators, including a pathologist, blinded to the patient's clinical features. The intensity of the HOXB13 staining was classified into one of four grades (0, absent; 1, weak; 2, intermediate; and 3, strong). Tumors with less than 5% HOXB13 expression were considered negative regardless of the intensity described [42]. This approach has been previously determined to be reliable and reproducible by several groups [43-45].

### Cell culture

Human PCa cell lines, LNCaP, PC3, and DU145, were routinely cultured in RPMI media (Invitrogen) supplemented with 5% FBS at 37°C in an atmosphere containing 5% CO_2_. MDA PCa 2b cells were grown in BRFF-HPC1 medium (Athena Environmental Sciences, Inc., Baltimore, MD, USA) with 20% FBS. Wild type HCT116 (p21+/+) and HCT116 (p21-/-) were kindly provided by Dr. Bert Vogelstein and have been well described previously [46]. All cultures were fed with fresh medium every 3-4 days.

### Plasmids and reagents

The pFLAG-HOXB13 and pGL-ARE4-Luc have been previously described [17]. The p21-luc, incorporating a 2.4 kb genomic fragment from the p21 promoter was kindly provided by Dr. Vogelstein. The pFLAG-p21 vector was constructed by PCR cloning. The pCMV-E2F1 was kindly provided by Dr. Hong-Wu Chen (UC Davis). The pE2F1-luc was provided by Dr. Chihuei Wang (Kaohsiung Medical University, Taiwan). pCMV-Hoxa13 and pCDNA-Hoxd13 were constructed from pMiw-Hoxa13 and pBK-Hoxd13, respectively, both of which were of mouse origin and provided by Dr. Atsushi Kuroiwa (Nagoya University, Japan). Antibodies to p16, p18, p21, p27, CDK2, CDK4, CDK6, CDK8, Cyclin D1, D2, D3, E, p107, RB, p130, E2F1-5, DP1-2, the androgen receptor and β-actin were from Santa Cruz Biotechnology. Anti-FLAG M5 antibodies and doxycycline were from Sigma. pTet-on, pTRE-luc, and pTRE-puro were from BD Biosciences. Synthetic testosterone, R1881, was from NEN Life Science (Boston, MA, USA) and used at a final concentration of 10 nM. Both charcoal dextran-treated (CDT) FBS and tetracycline-free FBS were from Invitrogen.

### Transient transfections

Approximately 1 × 10^5 ^cells were plated in a 24-well plate 16 hours before transfection. To determine the hormone effects, cells were grown under 5% charcoal dextran-treated (CDT) FBS for three days before the transfection. Transfections were carried out using Lipofectamine 2000 (Invitrogen) with 0.1 μg of reporter, 0.1 μg of test plasmid, and 2 ng of ranilla as described by the manufacturer's protocol. Six hours after transfection, the cells were washed and fed with medium containing 5% CDT-FBS. If needed, the cells were treated with either 10 nM R1881 synthetic androgen or ethanol. After 36 hours, the cells were washed with PBS, lysed with 100 μl of passive lysis buffer, and assayed for luciferase activity as relative light units (RLU) using a dual luciferase assay system (Promega). Transfection experiments were performed in triplicate and the results are reported as mean ± S.D.

### Stable transfections

Using the Tet-On system (BD Biosciences), the LNCaP PCa cells were transfected with Tet-on and a single clone was obtained by ring cloning and G418 selection (600 μg/ml; Invitrogen). Inducibility of the resulting clones was tested by transient transfection using pTRE-luc in the presence of doxycycline (Sigma). A second transfection was performed with pTRE-puro expressing FLAG-tagged HOXB13, followed by ring cloning and puromycin selection (1 μg/ml). The two best clones in terms of growing ability and protein inducibility were selected, namely S2 and S4. The Tet-on transfected cells were used as control cells. To suppress the HOXB13 in the LNCaP cells, short hairpin oligonucleotides were generated from either the 5' or 3' end of the HOXB13 [17]. The DNAs were ligated into the H1 RNA gene promoter-based vector, pSUPER.retro (DNA Engine, Seattle, WA, USA). The resulting vector was infected into PA316 packaging cells to produce retroviral supernatants. After infection of the LNCaP cells, viral plaques were isolated by a ring clone and selected by 1 μg/ml puromycin.

### Western Blotting Assay

Cells were grown to 50% confluence in P60 culture dishes containing 5% FBS-RPMI media. If necessary, the cells were grown in RPMI media containing either 5% tet-free FBS or CDT-FBS. The cells were then lysed in protein extraction buffer (1x TBS, 1% NP-40, 0.5% sodium deoxycholate, 0.1% SDS and protease inhibitors) followed by needle sonication to break up the ribonucleosome. Twenty μg of total cell lysates were loaded onto 10% Bis-Tris gel (Invitrogen) and separated using a Biorad electroporation system. After the proteins were transferred to a PVDF membrane, the primary antibodies were applied, followed by incubation with horse peroxidase-conjugated secondary antibodies. The blots were developed using the ECL detection system (Pierce).

### RT-PCR

RNA extraction from the cells was performed as previously described [47]. RT-PCR was performed to verify the expression of HOXB13, E2F1, and β-actin. The primer sequences for each gene were as follows: HOXB13fw, 5'-ccccactgagtttgccttctatc-3'; HOXB13rv, 5'-gcctcttgtccttggtgatgaac-3'; E2F1fw, 5'-aggccgccatccaggaaaag-3'; E2F1rv, 5'-ggatgccctcaacgacgttg -3'; β-actinfw, 5'-gcaccacaccttctacaatgagc-3'; β-actinrv, 5'-tagcacagcctggatagcaacg-3'.

### Growth Assays

The MTT *in vitro *proliferation assay was performed as previously described [17]. Briefly, the cells were grown in RPMI media containing 5% CDT-FBS for three days and then plated on 24-well plates at 30% confluence. The next day, if needed, the cells were treated with 100nM doxycycline and/or 1nM R1881 and grown for up to 7 days. Then, the cells were stained with 100 μl of 5 mg/ml MTT solution and incubated for 2 hours at 37°C. The reaction was stopped by adding 400 μl of extraction buffer (50% formamide and 10% SDS, pH 4.7). After overnight incubation at 37°C, the absorbance at 570 nm was measured using a microplate reader with SOFTmax PRO software (Molecular Devices, Sunnyvale, CA, USA). Densitometric values were analyzed with the Student's *t *test, using GraphPad Prism software (San Diego, CA, USA).

## List of Abbreviations Used

PCa: prostate cancer; AR: androgen receptor; AI: androgen-independent; AD: androgen-dependent; Dox: doxycyline; CDT: charcoal dextran-treated; ER: estrogen receptor; DAB: diaminobenzidine; RLU: relative light units; MTT: 3-(4,5-dimethylthiazol-2-yl)-2,5-diphenyltetrazolium bromide.

## Competing interests

The authors declare that they have no competing interests.

## Authors' contributions

YRK performed experiments and data analysis; KJO provided patient samples and evaluated data; RYP and NTXN performed experiments; DDK and TWK evaluated patient information; CC, KIN and KYA contributed to immunohistochemical scoring; MSK provided statistical assistance; CJ performed experiments, data analysis and wrote the manuscript. All authors read and approved the final manuscript.

## Supplementary Material

Additional file 1 Hormone-refractory prostate cancer patients cohort. Click here for file

Additional file 2 Hormone-dependent prostate cancer patients. Click here for file
